# Sociodemographic and geographic inequalities in diagnosis and treatment of older adults’ chronic conditions in India: a nationally representative population-based study

**DOI:** 10.1186/s12913-023-09318-6

**Published:** 2023-04-03

**Authors:** Sanjay K. Mohanty, Abhilasha, Radhe Shyam Mishra, Ashish Kumar Upadhyay, Owen O’Donnell, Jürgen Maurer

**Affiliations:** 1 Department of Population and Development, International Institute for Population Sciences, Govandi Station Road, Deonar, Mumbai India; 2 International Institute for Population Science, R4D India Project, Mumbai, India; 3 International Institute for Population Science, Research Coordinator, R4D India Project, Mumbai, India; 4 Professor of Applied Economics, Erasmus School of Economics, Erasmus School of Health Policy and Management, Erasmus University Rotterdam, Rotterdam, Netherlands; 5 Department of Economics, Institute of Health Economics and management, University of Lausanne, Lausanne, Switzerland

**Keywords:** Chronic condition, Non-communicable disease, Treatment, Inequality, LASI, India

## Abstract

**Context:**

Expeditious diagnosis and treatment of chronic conditions are critical to control the burden of non-communicable disease in low- and middle-income countries. We aimed to estimate sociodemographic and geographic inequalities in diagnosis and treatment of chronic conditions among adults aged 45 + in India.

**Methods:**

We used 2017–18 nationally representative data to estimate prevalence of chronic conditions (hypertension, diabetes, lung disease, heart disease, stroke, arthritis, cholesterol, and neurological) reported as diagnosed and percentages of diagnosed conditions that were untreated by sociodemographic characteristics and state. We used concentration indices to measure socioeconomic inequalities in diagnosis and lack of treatment. Fully adjusted inequalities were estimated with multivariable probit and fractional regression models.

**Findings:**

About 46.1% (95% CI: 44.9 to 47.3) of adults aged 45 + reported a diagnosis of at least one chronic condition and 27.5% (95% CI: 26.2 to 28.7) of the reported conditions were untreated. The percentage untreated was highest for neurological conditions (53.2%; 95% CI: 50.1 to 59.6) and lowest for diabetes (10.1%; 95% CI: 8.4 to 11.5). Age- and sex-adjusted prevalence of any diagnosed condition was highest in the richest quartile (55.3%; 95% CI: 53.3 to 57.3) and lowest in the poorest (37.7%: 95% CI: 36.1 to 39.3). Conditional on reported diagnosis, the percentage of conditions untreated was highest in the poorest quartile (34.4%: 95% CI: 32.3 to 36.5) and lowest in the richest (21.1%: 95% CI: 19.2 to 23.1). Concentration indices confirmed these patterns. Multivariable models showed that the percentage of untreated conditions was 6.0 points higher (95% CI: 3.3 to 8.6) in the poorest quartile than in the richest. Between state variations in the prevalence of diagnosed conditions and their treatment were large.

**Conclusions:**

Ensuring more equitable treatment of chronic conditions in India requires improved access for poorer, less educated, and rural older people who often remain untreated even once diagnosed.

**Supplementary Information:**

The online version contains supplementary material available at 10.1186/s12913-023-09318-6.

## Highlights

• Little is known about sociodemographic and geographic inequalities in the diagnosis and treatment of chronic illness among middle-aged and older adults in India.

• Self-reported diagnosed chronic conditions are more prevalent among socially advantaged groups but the disadvantaged are more likely to be untreated.

• To ensure more equitable treatment of chronic conditions, efforts to improve access should be directed towards poorer, less educated, and ruralolder people.

**Supplementary Information:**

The online version contains supplementary material available at 10.1186/s12913-023-09318-6.

## Introduction

Chronic non-communicable diseases (NCDs) are the leading causes of mortality, disability, hospitalisation, and catastrophic health expenditure worldwide [1–4] and in India [1, 5]. Low- and middle-income countries (LMICs) account for over three fourth of NCD deaths and over four fifth of all premature NCD deaths [1, 6]. Increasing longevity, along with earlier onset of NCDs, has increased the chronic disease burden in these countries [7, 8]. In 2016, the burden of NCDs were greater than that of communicable diseases in every state of India [9]. NCDs can cause long term economic hardship for individuals and their families, which, in turn, can lead to treatment non-adherence and so heightened health risks [10].

Prevention and control of chronic diseases are important public health priorities of national governments and development partners [11, 12]. Inexpensive generic drugs can manage health effects of chronic conditions and reduce premature mortality such as ischemic heart disease, stroke, and diabetes. Early diagnosis and treatment can prevent the emergence of complications and so reduce the disease burden and its economic cost [13].

Despite the availability of treatments to control chronic diseases that are diagnosed sufficiently early, rates of diagnosis and treatment often remain low in LMICs. For example, around half of older Chinese adults (45 +) with an indication of diabetes have been estimated to be undiagnosed and about one third of the diagnosed cases were not on medication [14]. The pattern was similar for other chronic conditions. In India, about 21% of older adults (45 +) who reported having been diagnosed with high blood pressure (HBP) or who were measured to have HBP were estimated to be untreated [15] and there seems to be improvement in hypertension management [16, 17]. Analysis of earlier data revealed that over 90% of older adults (50 +) in India, China, and Ghana (and about 70% in South Africa) who appeared to have symptoms of depression did not receive treatment [17]. In Mexico, over two fifths of the older adults (50 +) who reported being diagnosed with one or more chronic condition were untreated for at least one condition [18].

Unmet need for chronic disease treatment may be due to lack of awareness, access, and affordability of healthcare facilities that deliver the appropriate treatment [19]. Lower education groups may particularly lack awareness [20, 21]. Poorer individuals are least able to afford care that must be paid for directly or indirectly through travel costs and lost earnings. For these reasons, socially disadvantaged groups are expected to have the highest levels of unmet need. For cardiovascular conditions, unmet need for treatment tends to be higher among women [22, 23].

Estimates from over 1.3 million adults in India suggests high prevalence of diabetes and hypertension across all regions and socio-economic groups [24]. About one-third of older adults have multiple chronic conditions with strong age and socio-economic gradient [25]. Risk factors of many of these chronic diseases have been increasing and shows large regional variation in the country [26]. There is also some evidence that the prevalence of untreated chronic conditions is higher among younger adults than older adults [7, 21, 24–27].

The awareness, treatment and control of hypertension and diabetes in India is low, with variation across gender, socioeconomic conditions and states [15, 17, 28–34]. Among those aged 45 years and older with hypertension, 54.4%, 50.8% and 28.8% were aware, treated, and controlled, respectively [15]. Among all diabetic individuals aged 15–49 years, 53% were aware, 41% were treated and 25% were controlled [31]. Among individuals with both hypertension and diabetes, only 29% were aware, 17% were treated and 4% were controlled [34]. Only 43% of people diagnosed with tuberculosis (TB) in India completed treatment and only 7% completed treatment for multi-drug resistant TB [35]. Untreated morbidity was higher among those who were elderly, poor and living alone [20].

In India, the cost of treatment of chronic diseases, including maintenance medication, is high and often a major cause of high medical spending and financial catastrophe [5]. A stated aim of the National Health Policy is to reduce the health and financial burden of chronic conditions [11]. Studies that have sought to deliver evidence relevant to the realisation of this aim have largely focused on the prevalence and risk factors of chronic disease and their financial implications for households. There is a lack of population-based estimates of the socioeconomic and geographical variations in the treatment for chronic diseases among older adults in India. This paper aims to help fill this gap by estimating the extent to which seven diagnosed chronic conditions reported by older adults remain untreated and how this varies across sociodemographic groups.

## Data and methods

### Data

We used data from the first (as yet, only) wave of the Longitudinal Ageing Study in India (LASI) conducted in 2017–18. The study used stratified, multistage probability cluster random sampling to select 73,396 individuals aged 45 + and their spouses from 43,584 sampled households. The survey was conducted in all the states and union territories of the country. The sample was representative of the non-institutionalized population aged 45 + (and their spouses) at the state level as well as nationally. The response rate was 95.8% at the household level and 87.3% at the individual level. The sampling design is described in more detail in the LASI Report [36] and other publications [7, 37]. LASI obtained ethical approval from the Health Ministry’s Screening Committee (Government of India) and the Institutional Review Boards at IIPS and its collaborating institutions. Written informed consent was obtained from all study participants. The LASI data are publicly available and can be accessed through registration at https://www.iipsindia.ac.in/content/lasi-publications.

### Measures

We used data on self-reported diagnoses of seven major chronic conditions. Specifically, the study participants were asked whether a health professional had ever told them they have i) hypertension or high blood pressure, ii) diabetes or high blood sugar, iii) chronic lung disease, such as asthma, chronic obstructive pulmonary disease/chronic bronchitis, or other chronic lung problems, iv) chronic heart diseases, such as coronary heart disease (heart attack or myocardial infarction), congestive heart failure, or other chronic heart problems, v) stroke, vi) arthritis or rheumatism, osteoporosis, or other bone/joint diseases, vii) any neurological or psychiatric problems, such as depression, Alzheimer’s/dementia, unipolar/bipolar disorders, convulsions, Parkinson’s, etc., viii) cholesterol, and ix) any other chronic condition (thyroid disease, skin disease, gastrointestinal etc.). A positive answer for each one of the conditions, except ix), prompted a question about any treatment received. For example, if a participant reported having been diagnosed with hypertension, they were asked: “In order to control your blood pressure or hypertension, are you currently taking any medication or are you under salt/diet restriction?” See S1 Table for the respective questions about treatment of the other conditions. From these questions, we defined a binary indicator of any diagnosed chronic condition (no = 0, yes = 1) and, for those with such a condition, an indicator of the proportion of the participant’s conditions that were untreated (0 = none untreated,1 = all untreated). Since there was no follow-up question about medication for ix) other chronic conditions, we did not include reports on this category.

Household monthly per capita consumption expenditure (MPCE) was used as the primary indicator of socioeconomic status. This measure is used in India to identify those in poverty. We constructed MPCE from detailed reports of items of expenditure and consumption, including goods produced by the household, excluding expenditure on healthcare and medicines (see S1 Text for details).

### Statistical analysis

To estimate the prevalence of any diagnosis of chronic condition, we restricted the sample to participants aged 45 years and older. Using the same sample, we estimated prevalence of each of the conditions. In this analysis, we defined heart disease and stroke as one condition because of small numbers and the common aetiology of these conditions. We used a sub-sample of participants who reported having at least one chronic condition to estimate the untreated fraction of all diagnosed chronic conditions. We estimated the prevalence of any diagnosis of chronic condition and the untreated fraction of all diagnosed chronic conditions, by state, by MPCE quartile groups, and by other sociodemographic characteristics (years of schooling, age group, sex, urban–rural residence, caste, religion, marital status, living arrangement, employment status, and health insurance status). We adjusted the estimates by state and sociodemographic groups for age and sex differences using the age-sex composition of the nationally representative full sample as the reference.

At the state level, we used a scatter plot and fitted a linear regression to examine the association between prevalence of any diagnosed chronic condition and economic development measured by net state domestic product (NSDP) per capita. Similarly, we examined the association between the untreated fraction of all diagnosed chronic conditions and NSDP per capita. We excluded three union territories (Dadra and Nagar Haveli, Daman and Diu, and Lakshadweep) from these analyses due to the unavailability of NSDP per capita for the reference period 2017–2018.

We used concentration curves to assess relative inequality in prevalence of any reported diagnosed chronic condition/untreated fraction of all diagnosed chronic conditions along the full distribution of MPCE. Each curve shows the cumulative proportion of the outcome against the cumulative rank (from poor to rich) by MPCE [38]. We used dominance analysis to test whether the concentration curve deviated significantly from the 45-degree line and so the null hypothesis of equality could be rejected [38]. To quantify the degree of socioeconomic inequality in prevalence of any diagnosed chronic condition and the untreated fraction of all diagnosed chronic conditions along the full distribution of MPCE, we used concentration indices, i.e., the scaled covariance between each outcome and rank in the MPCE distribution [39]. A positive (negative) concentration index indicates a higher prevalence/untreated fraction among richer (poorer) individuals. We adjusted the concentration indices for age and sex.

We used multivariable probit regression to estimate fully adjusted marginal effects of MPCE quartile groups, other sociodemographic characteristics, and state on the probability of any diagnosed chronic conditions. We used multivariable fractional regression [40] to estimate respective fully adjusted marginal effects of the untreated fraction of all diagnosed chronic conditions. Effectively, this was a generalized linear model with a probit link function used to fit the conditional mean of the outcome and a binomial family for its distribution, with robust standard errors. Each marginal effect was averaged over the sample used in the respective regression.

In all our analyses, we applied sampling weights and took account of stratification and cluster sampling to estimate confidence intervals (CIs). All the analyses were done using Stata 15.0.

## Results

**Fig. 1:**
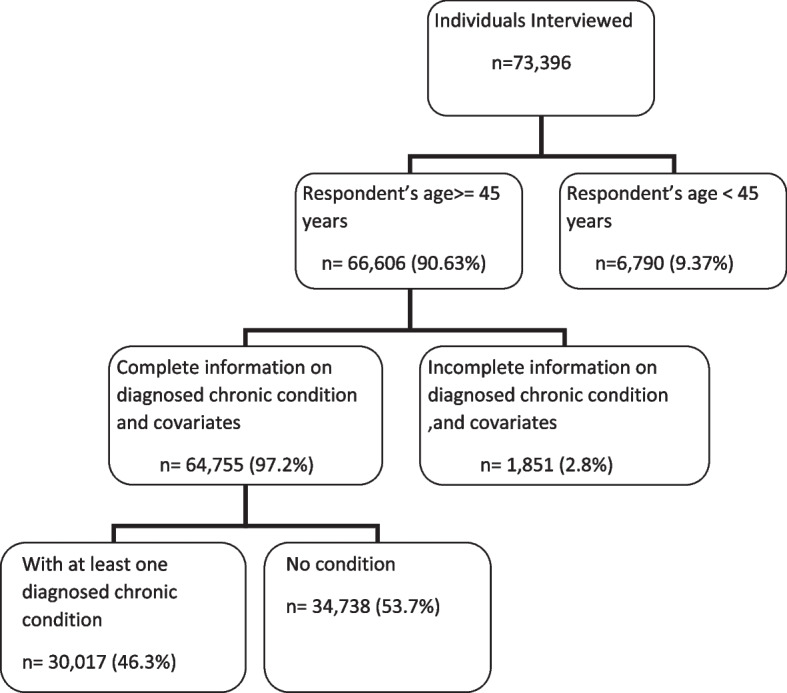
Flow chart for sample size

Figure 1 shows the construction of our analysis sample. Of the 73,396 participants interviewed, 6,790 (9.4%) participants were below age 45 years and hence excluded from the analysis. Of the remaining 66,606 participants, 1,851 (2.8%) participants had missing information on any of the seven chronic conditions or their covariates. Among the 64,755 participants with complete information on the conditions and their covariates, 30,017 reported having at least one diagnosed condition and these participants were included in the sub-sample used to estimate and analyse the untreated fraction of all diagnosed chronic conditions.

Table 1 shows the characteristics of the analytical sample of this study. Among those with at least one condition that were used to estimate the proportion of untreated conditions, a majority of participants was female (56.8%). About 46.7% of this analysis sample had no formal schooling. A large majority were living in rural areas (62.5%) and were currently married (71.1%). About 36.2% of participants with at least one condition were working and little more than one fifth had health insurance (21.4%).

**Table 1:** Descriptive statistics of analyses samples, adults aged 45 years and older in India, LASI 2017–18

	**Sample for diagnosed chronic condition prevalence**		**Sample for untreated diagnosed chronic conditions**	
	***N* = 64,755**		***N* = 30,017**	
	**Frequency**	**Percent**	**Frequency**	**Percent**
**MPCE Quartile**				
First	14,153	25.0	5,203	20.8
Second	14,460	25.0	6,304	23.6
Third	16,987	25.0	8,250	26.1
Fourth	19,155	25.0	10,260	29.5
**Educational attainment**				
Illiterate	30,558	50.9	12,981	46.7
Less than 5 years	7368	11.0	3,514	11.7
5–9 years completed	14,684	20.4	7,067	21.3
10 years or more	12,145	17.7	6,455	20.3
**Age**				
45–54	23,897	34.9	8,769	27.8
55–64	19,895	30.1	9,448	30.6
65–74	14,309	23.7	7,998	27.8
75 +	6654	11.3	3,802	13.9
**Sex**				
Male	30,220	46.2	13,021	43.2
Female	34,535	53.8	16,996	56.8
**Residence**				
Rural	42,315	69.8	17,401	62.5
Urban	22,440	30.2	12,616	37.5
**Caste**				
Scheduled Tribes	11,407	8.7	3,665	5.4
Scheduled Castes	10,812	19.6	4,789	18.5
OBC	24,555	45.1	11,859	46.3
Others	17,981	26.6	9,704	29.8
**Religion**				
Hindu	47,403	82.2	21,691	80.2
Muslim	7531	11.3	4,118	13.0
Christian	6495	2.9	2,458	2.7
Others	3326	3.5	1,750	4.1
**Marital Status**				
Currently married	48,229	73.5	21,452	71.1
Currently not married	16,526	26.5	8,565	28.9
**Working status**				
Currently working	29,754	46.7	10,550	36.2
Ever worked but currently not working	16,972	27.6	9,841	34.5
Never worked	18,029	25.7	9,626	29.3
**Health Insurance**				
No	50,107	79.7	23,201	78.6
Yes	14,648	20.3	6,816	21.4

The sample described in the two columns to the left includes those with complete data on chronic conditions and covariates and was used to estimate prevalence of conditions. The sample described in the columns to the right includes those with at least one reported diagnosed condition and was used to estimate the proportion of untreated conditions. Frequencies are unweighted. Sample percentages are weighted

Table 2 shows estimates of the prevalence of diagnosis of at least one chronic condition as well as the percentage of untreated conditions among those with any condition, and age-sex adjusted estimates of these outcomes by sociodemographic characteristics. The prevalence of any diagnosed chronic disease was estimated to be 46.1% (95% CI 44.9 to 47.3) among adults aged 45 years and over in India. This prevalence increased strongly with age and was higher among females (48.9%: 95% CI 47.6 to 50.1) than males (42.9%: 95% CI 41.6 to 44.3). Adjusted for age and sex, the estimated prevalence increased monotonically when moving from the poorest quartile (37.7%: 95% CI 36.1 to 39.3) to the richest one (55.3%: 95% CI 53.3 to 57.3). Prevalence was estimated to increase from 39.5% (95% CI 38.1 to 40.9) among individuals in the lowest educational group to 57.6% (95% CI 55.6 to 59.7) among individuals with the highest education level. Prevalence was also higher in urban areas (57.6%: 95% CI 55. to 59.6) than in rural areas (41.2%: 95% CI 40.1 to 42.3). Average number of chronic conditions was also higher in groups that were richer, better educated, older, female, and urban (S2 Table).

**Table 2:** Age-sex adjusted estimates of prevalence of any diagnosed chronic condition and untreated fraction of all diagnosed chronic conditions by socioeconomic and demographic characteristics, adults aged 45 years and older in India, LASI 2017–18

	**Any diagnosed chronic condition (*N* = 64,755)**	**Untreated fraction of all diagnosed chronic conditions (*N* = 30,017)**
	**Percent (95% CI)**	**Percent (95% CI)**
**India**	**46.1 (44.9, 47.3)**	**27.5 (26.2, 28.7)**
**MPCE Quartile**		
First	37.7 (36.1, 39.3)	34.4 (32.3, 36.5)
Second	43.4 (41.9, 44.9)	30.1 (28.1, 32.1)
Third	48.1 (46.5, 49.8)	26.7 (25.2, 28.3)
Fourth	55.3 (53.3, 57.3)	21.1 (19.2, 23.1)
F, statistic (*p*, value)	< 0.001	< 0.001
**Educational attainment**		
Illiterate	39.5 (38.1, 40.9)	32.6 (31.0, 34.2)
Less than 5 years	49.3 (47.4, 51.3)	27.8 (25.7, 30.0)
5–9 years completed	51.3 (49.5, 53.0)	24.0 (22.1, 25.9)
10 years or more	57.6 (55.6, 59.7)	19.6 (17.9, 21.4)
F, statistic (*p*, value)	< 0.001	< 0.001
**Age**		
45–54	36.6 (34.8, 38.4)	30.4 (27.9, 32.8)
55–64	46.9 (45.1, 48.7)	27.7 (26.1, 29.2)
65–74	54.1 (52.2, 56.0)	24.8 (23.1, 26.4)
75 +	56.8 (54.3, 59.4)	26.6 (23.9, 29.3)
F, statistic (*p*, value)	< 0.001	< 0.001
**Sex**		
Male	42.9 (41.6, 44.3)	28.4 (27.0, 29.8)
Female	48.9 (47.6, 50.1)	26.8 (25.4, 28.1)
	< 0.001	0.0324
**Residence**		
Rural	41.2 (40.1, 42.3)	32.2 (31.0, 33.5)
Urban	57.6 (55.6, 59.6)	19.5 (17.7, 21.3)
F, statistic (*p*, value)	< 0.001	< 0.001
**Caste**		
Scheduled Tribes	29.1 (26.8, 31.4)	35.9 (32.3, 39.6)
Scheduled Castes	43.7 (42.0, 45.5)	30.8 (28.5, 33.1)
OBC	47.3 (45.3, 49.2)	27.2 (25.2, 29.2)
Others	51.5 (49.9, 53.1)	24.2 (22.9, 25.6)
F, statistic (*p*, value)	< 0.001	< 0.001
**Religion**		
Hindu	44.9 (43.6, 46.3)	28.5 (27.2, 29.9)
Muslim	53.6 (49.3, 58.0)	22.2 (18.7, 25.6)
Christian	42.2 (35.8, 48.7)	26.6 (23.5, 29.8)
Others	53.0 (49.6, 56.5)	23.6 (20.4, 26.8)
F, statistic (*p*, value)	< 0.001	< 0.001
**Marital Status**		
Currently marries	46.6 (45.0, 48.2)	27.4 (26.0, 28.8)
Currently not married	44.8 (43.4, 46.2)	27.6 (25.8, 29.4)
F, statistic (*p*, value)	0.127	0.843
**Working status**		
Currently working	37.6 (36.1, 39.1)	30.7 (29.0, 32.5)
Ever worked but currently not working	54.8 (53.1, 56.5)	26.0 (24.2, 27.7)
Never worked	52.0 (49.9, 54.2)	25.1 (23.0, 27.2)
F, statistic (*p*, value)	< 0.001	< 0.001
**Health Insurance**		
No	45.2 (44.1, 46.4)	27.8 (26.6, 29.1)
Yes	49.6 (47.5, 51.7)	26.0 (24.1, 28.0)
F, statistic (*p*, value)	< 0.001	0.064

We estimated that 27.5% (95% CI 26.2 to 28.7) of the diagnosed chronic conditions were untreated. The percentage of untreated conditions was highest in the poorest quartile (34.4%: 95% CI 32.3 to 36.5) and lowest in the richest quartile (21.1%: 95% CI 19.2 to 23.1). It was highest among those with no schooling (32.6%: 95% CI 31.0 to 34.2) and decreased monotonically as education increased. About one third of the diagnosed conditions were untreated among those living in rural areas (32.2%: 95% CI 31.0 to 33.5) as against only one fifth among those living in urban areas (19.5%: 95% CI 17.7 to 21.3). The percentage of untreated conditions was slightly higher among males (28.4%: 95% CI 27.0 to 29.8), scheduled tribes (35.9%: 95% CI 32.3 to 39.6), the employed (30.7%: 95% CI 29.0 to 32.5), and those without health insurance (27.8%: 95% CI 24.1 to 28.0) compared with their respective counterparts.

Prevalence varied considerably by type of condition from 2.3% (95% CI 2.0 to 2.5) for cholesterol to 27.3% (95% CI 26.2 to 28.2) for hypertension (Table 3). Age-sex adjusted prevalence of each chronic condition by sociodemographic characteristics are given in appendix (S3 Table).

**Table 3:** Adjusted prevalence and concentration index for of any diagnosed chronic condition and untreated fraction of all diagnosed chronic conditions, adults aged 45 years and older in India

	**Any diagnosed chronic condition (*N* = 64,755)**		**Untreated fraction of all diagnosed chronic conditions**		
	**Prevalence (95% CI), %**	**Concentration Index (95% CI)**	***N***	**Untreated fraction (95% CI), %**	**Concentration Index (95% CI)**
Any conditions	46.1 (44.9, 47.3)	0.078 (0.067, 0.090)	30,017	27.5 (26.2, 28.7)	-0.059 (-0.071, -0.047)
Hypertension	27.3 (26.2, 28.2)	0.073 (0.060, 0.086)	18,730	16.2 (14.8, 17.1)	-0.047 (-0.060, -0.034)
Diabetes	12.1 (11.1, 13.1)	0.056 (0.043, 0.068)	8,282	10.1 (8.4, 11.5)	-0.037 (-0.052, -0.022)
Lung disease	6.7 (5.9, 7.4)	0.007 (-0.005, 0.018)	3,635	31.1 (25.4, 34.0)	-0.059 (-0.087, -0.031)
Heart disease/ stroke	5.5 (5.0, 5.9)	0.014 (0.008, 0.021)	3,364	32.5 (29.3, 36.2)	-0.080 (-0.112, -0.049)
Cholesterol	2.3 (2.0, 2.5)	0.015 (0.012, 0.018)	2231	39.3 (34.7, 43.8)	-0.030 (-0.069, 0.009)
Arthritis	16.1 (15.2, 16.9)	0.015 (0.005, 0.025)	9,420	44.9 (43.6, 47.4)	-0.037 (-0.056, -0.018)
Neurological	2.4 (2.1, 2.7)	0.002 (-0.001, 0.005)	1,454	53.2 (50.1, 59.6)	-0.038 (-0.092, 0.016)

1) Any neurological, or psychiatric problems includes depression, Alzheimer’s/Dementia, unipolar/bipolar disorders, convulsions, Parkinson’s etc.

The probability of not receiving treatment also varied substantially by condition (Table 3). While prevalence was lowest for cholesterol, neurological problems were the most likely to remain untreated (53.2%: 95% CI 50.1 to 59.6) At the other extreme, we estimated that only about 10.1% (95% CI 8.4 to 11.5) of those who reported being diagnosed with diabetes were not receiving any treatment. For each type of condition, individuals who were poorer, less educated, rural dwellers, in scheduled tribes, and working were more likely to be untreated (Table 4).

**Table 4:** Age sex adjusted estimates of fraction untreated (%) for each diagnosed condition, adults aged 45 years and older in India, LASI 2017-18

	*N* = 8,282	*N* = 3,635	*N* = 3,364	2,231	*N* = 9,420	*N* = 1,454	
	**Hypertension**	**Heart disease/stroke**	**Diabetes**	**Lung disease**	**Cholesterol**	**Arthritis**	**Any Neurological problems**
**India**	16.2 (14.8, 17.1)	10.1 (8.4, 11.5)	31.1 (25.4, 34.0)	32.5 (29.3, 36.2)	39.3 (34.7, 43.8)	44.9 (43.6, 47.4)	53.2 (50.1, 59.6)
**MPCE Quintile**							
1st quartile	21.6 (19.5, 24.0)	43.4 (37.4, 49.4)	16.5 (12.7, 20.2)	36.3 (30.9, 41.7)	44.3 (31.9, 56.7)	48.5 (44.8, 52.2)	65.0 (57.3, 72.8)
2nd quartile	18.9 (16.9, 20.8)	35.6 (30.3, 40.9)	11.3 (8.6, 14.0)	31.7 (26.8, 36.7)	43.8 (35.0, 52.7)	47.3 (43.2, 51.4)	50.6 (42.1, 59.0)
3rd quartile	15.0 (13.5, 16.7)	33.2 (28.4, 38.0)	9.5 (7.6, 11.3)	30.7 (26.0, 35.4)	36.9 (28.9, 44.9)	46.2 (42.8, 49.6)	51.0 (44.4, 57.5)
4th quartile	11.4 (9.5, 13.4)	23.7 (18.2, 29.2)	7.1 (4.9, 9.3)	21.0 (14.7, 27.3)	37.7 (32.6, 42.8)	40.4 (37.7, 43.1)	53.6 (45.6, 61.6)
F, statistic (*p*, value)	< 0.001	< 0.001	< 0.001	< 0.001	< 0.001	0.003	0.002
**Educational attainment**							
Illiterate	20.7 (19.2, 22.3)	40.1 (35.8, 44.4)	14.9 (12.3, 17.5)	34.7 (30.8, 38.6)	45.7 (37.2, 54.2)	47.0 (44.1, 49.9)	57.7 (51.3, 64.2)
Less than 5 years	15.7 (13.3, 18.0)	34.7 (27.0, 42.3)	12.1 (9.0, 15.1)	30.9 (24.7, 37.2)	35.6 (25.8, 45.4)	46.0 (41.7, 50.2)	46.0 (35.3, 56.7)
5, 9 years completed	13.1 (11.4, 15.0)	32.1 (25.8, 38.3)	7.2 (5.4, 9.0)	25.5 (20.5, 30.4)	41.1 (34.0, 48.3)	43.0 (39.3, 46.7)	52.2 (43.9, 60.5)
10 years or more	10.0 (8.0, 12.1)	20.7 (14.6, 26.9)	6.4 (4.3, 8.6)	18.1 (9.5, 26.8)	35.4 (29.0, 41.7)	43.1 (38.6, 47.6)	56.8 (45.4, 68.3)
F, statistic (*p*, value)	< 0.001	< 0.001	< 0.001	< 0.001	< 0.001	0.299	0.352
**Age**							
45, 54	20.9 (18.1, 23.9)	32.9 (26.7, 39.1)	9.8 (6.8, 12.9)	38.7 (34.0, 43.5)	46.4 (39.8, 53.0)	46.5 (43.4, 49.6)	49.2 (39.0, 59.4)
55, 64	15.7 (14.0, 17.4)	36.0 (31.8, 40.3)	10.5 (8.4, 12.6)	27.4 (20.1, 34.6)	40.2 (34.6, 45.9)	48.2 (43.4, 53.0)	50.6 (43.4, 57.7)
65, 74	12.8 (11.2, 14.4)	26.7 (20.1, 33.2)	8.7 (6.7, 10.8)	25.1 (19.2, 31.1)	34.5 (24.6, 44.4)	42.8 (38.6, 47.1)	55.0 (47.2, 62.9)
75 +	13.5 (11.5, 16.0)	39.9 (34.3, 45.6)	11.9 (8.1, 15.7)	31.6 (25.9, 37.3)	27.9 (10.3, 45.4)	43.5 (38.3, 48.7)	69.7 (59.7, 79.7)
F, statistic (*p*, value)	< 0.001	0.473	< 0.001	0.002	< 0.001	0.645	< 0.001
**Sex**							
Male	16.2 (14.7, 17.9)	32.6 (29.4, 35.8)	10.6 (8.8, 12.3)	29.3 (24.8, 33.9)	39.4 (33.1, 45.6)	52.0 (47.8, 56.3)	50.4 (44.4, 56.4)
Female	15.7 (14.4, 17.1)	32.9 (27.0, 38.8)	9.4 (7.6, 11.3)	30.0 (25.0, 34.9)	39.2 (33.3, 45.1)	41.6 (38.8, 44.5)	58.4 (51.7, 65.1)
F, statistic (*p*, value)	0.048	0.197	0.845	0.973	0.0324	0.002	0.059
**Residence**							
Rural	19.8 (18.7, 21.1)	40.9 (37.4, 44.4)	14.0 (12.1, 15.9)	32.8 (29.2, 36.3)	49.1 (43.5, 54.7)	47.9 (45.4, 50.4)	58.3 (53.1, 63.4)
Urban	10.5 (8.8, 12.3)	21.1 (16.8, 25.4)	6.3 (4.4, 8.2)	22.3 (15.9, 28.8)	32.6 (27.3, 37.9)	40.1 (37.4, 42.7)	47.4 (40.0, 54.8)
F, statistic (*p*, value)	< 0.001	< 0.001	< 0.001	< 0.001	< 0.001	< 0.001	0.004
**Caste**							
Scheduled Tribes	21.6 (17.7, 25.5)	45.5 (35.4, 55.7)	13.0 (8.1, 17.8)	34.7 (25.4, 43.9)	53.0 (33.2, 72.7)	58.0 (51.3, 64.7)	61.6 (48.7, 74.5)
Scheduled Castes	18.2 (15.9, 20.5)	40.1 (33.3, 47.0)	13.0 (9.9, 16.1)	31.4 (25.6, 37.2)	37.7 (25.6, 49.8)	46.3 (42.1, 50.5)	60.7 (52.2, 69.2)
OBC	15.7 (13.8, 17.7)	30.5 (25.5, 35.4)	8.7 (6.8, 10.6)	30.7 (24.7, 36.8)	40.6 (34.6, 46.5)	46.9 (44.3, 49.5)	54.4 (47.6, 61.1)
Others	14.1 (12.6, 15.6)	30.3 (26.3, 34.3)	10.2 (7.9, 12.5)	25.0 (20.4, 29.7)	37.5 (32.0, 43.1)	40.2 (37.2, 43.2)	50.0 (44.1, 55.9)
F, statistic (*p*, value)	< 0.001	0.054	0.002	0.104	< 0.001	< 0.001	0.095
**Religion**							
Hindu	17.0 (15.7, 18.4)	17.0 (15.7, 18.4)	10.4 (8.7, 12.1)	0.296 (0.250, 0.341)	38.0 (32.9, 43.2)	0.463 (0.443, 0.484)	55.8 (50.7, 60.9)
Muslim	12.3 (9.1, 15.6)	12.3 (9.1, 15.6)	8.9 (4.8, 13.1)	0.315 (0.250, 0.381)	38.8 (28.6, 48.9)	0.383 (0.340, 0.426)	45.3 (36.0, 54.5)
Christian	11.1 (8.2, 14.1)	11.1 (8.2, 14.1)	6.4 (3.1, 9.8)	0.253 (0.152, 0.353)	40.3 (26.8, 53.7)	0.600 (0.519, 0.681)	59.8 (41.9, 77.6)
Others	12.1 (9.7, 14.7)	12.1 (9.7, 14.7)	8.8 (4.8, 12.7)	0.282 (0.157, 0.406)	48.2 (40.4, 55.9)	0.398 (0.297, 0.500)	59.9 (43.9, 76.0)
F, statistic (*p*, value)	< 0.001	0.19	0.333	0.744	< 0.001	< 0.001	0.199
**Marital Status**							
Currently married	15.8 (14.4, 17.2)	34.4 (31.1, 37.8)	9.4 (7.8, 11.1)	0.309 (0.266, 0.352)	38.3 (33.4, 43.3)	0.453 (0.428, 0.477)	54.3 (48.6, 60.0)
Currently not married	16.2 (14.4, 18.2)	28.8 (22.0, 35.6)	11.6 (9.0, 14.3)	0.271 (0.207, 0.335)	42.5 (33.1, 51.8)	0.460 (0.428, 0.491)	56.0 (49.0, 62.9)
F, statistic (*p*, value)	0.701	0.138	0.131	0.214	0.8376	0.764	0.701
**Working status**							
Currently working	18.6 (16.7, 20.7)	39.9 (34.1, 45.7)	11.3 (9.0, 13.5)	0.335 (0.278, 0.393)	50.0 (42.3, 57.7)	0.482 (0.451, 0.514)	57.1 (51.0, 63.3)
Ever worked but currently not working	14.7 (13.0, 16.5)	32.0 (28.2, 35.9)	10.0 (7.5, 12.5)	0.286 (0.241, 0.331)	36.4 (29.5, 43.4)	0.437 (0.405, 0.469)	53.1 (46.3, 60.0)
Never worked	14.3 (12.4, 16.4)	27.8 (21.2, 34.4)	8.6 (6.2, 11.0)	0.265 (0.194, 0.336)	33.1 (25.8, 40.5)	0.444 (0.406, 0.482)	54.5 (45.4, 63.6)
F, statistic (*p*, value)	0.002	0.263	0.012	0.11	< 0.001	0.078	0.729
**Health Insurance**							
No	16.5 (15.4, 17.9)	33.4 (29.5, 37.2)	10.3 (8.6, 11.9)	0.302 (0.256, 0.349)	41.3 (36.5, 46.1)	0.455 (0.434, 0.476)	54.0 (49.3, 58.6)
Yes	13.6 (11.5, 15.5)	30.3 (25.0, 35.6)	8.9 (6.3, 11.5)	0.272 (0.223, 0.320)	32.9 (25.8, 40.0)	0.454 (0.421, 0.486)	57.9 (48.6, 67.1)
F, statistic (*p*, value)	0.003	0.283	0.333	0.236	0.1106	0.878	0.377

**Fig. 2:**
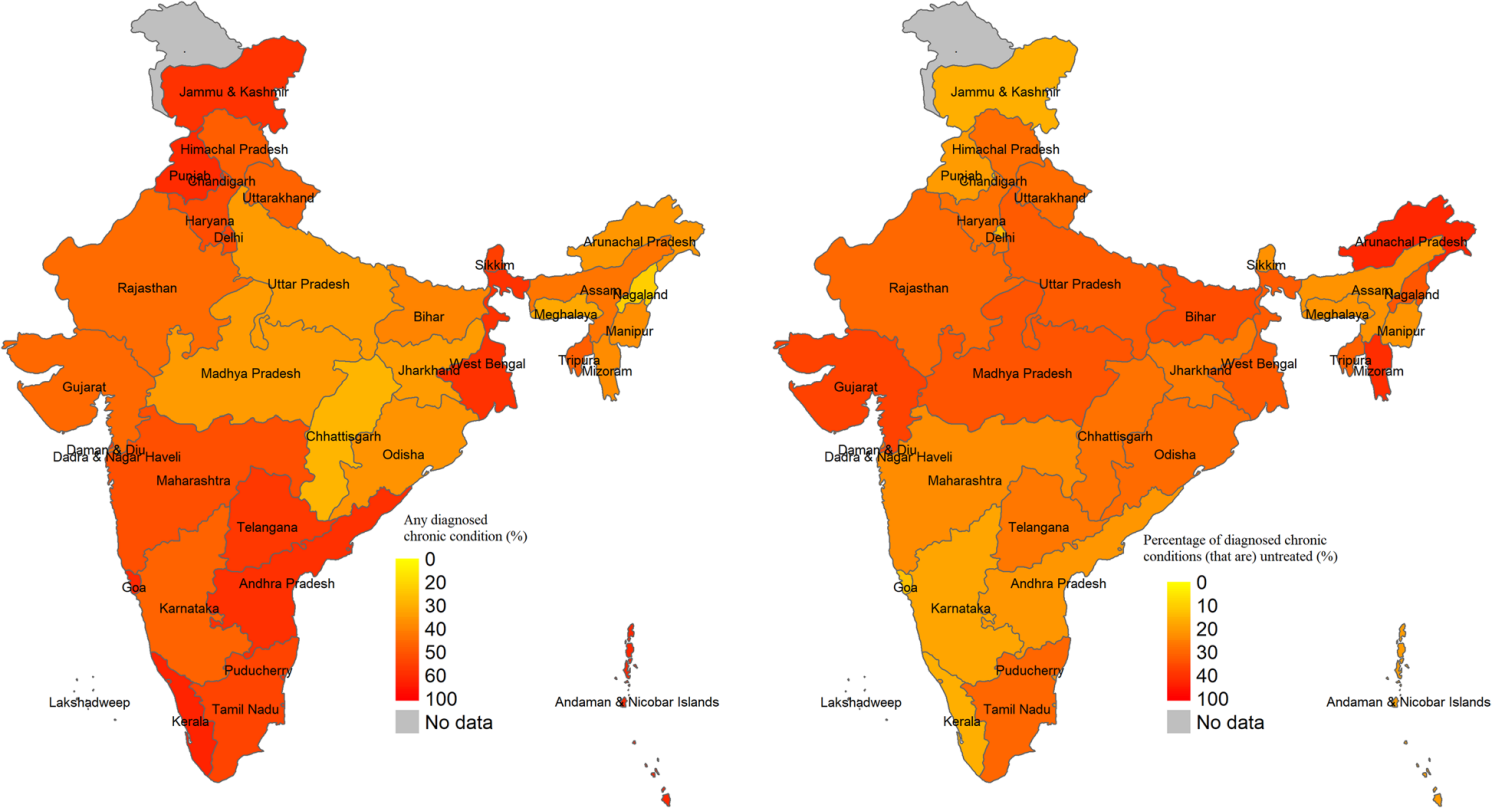
Adjusted estimates of prevalence of any diagnosed chronic condition and untreated fraction of all diagnosed chronic conditions by states, adults aged 45 years and over in India, LASI 2017–18. Note. Adjusted for age and sex. For numerical estimates see S4 Table

**Fig. 3:**
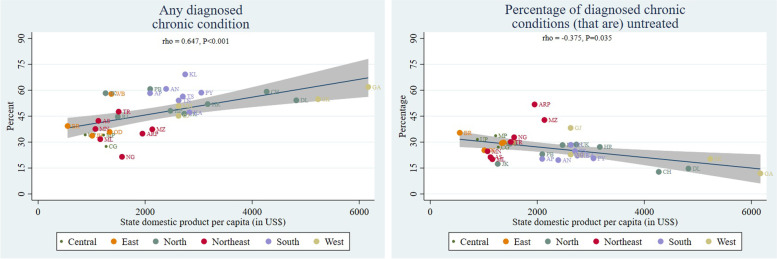
Association of prevalence of any diagnosed chronic condition and untreated fraction of all diagnosed chronic conditions with state domestic product per capita, adults aged 45 years and older LASI 2017–18

Figure 2 shows substantial geographical variation in the prevalence of any diagnosed chronic condition and the percentage of untreated conditions. States with a higher prevalence were those that are relatively more advanced in the demographic transition, such as Kerala (69.4%: 95% CI 66.5 to 72.2), Goa (61.9%: 95% CI 58.2 to 65.7), Punjab (60.7%: 95% CI 57.8 to 63.66), and Andhra Pradesh 58.3%: 95% CI 55.7 to 61.2). This is confirmed by Fig. 3 that shows a positive, but weak, association between the prevalence of any diagnosed condition and state NSDP per capita. States with a relatively lower prevalence tended to have a higher percentage of untreated conditions, although there are exceptions to this inverse relationship. More than half of the diagnosed conditions (51.8%: 95% 39.6 to 64.0) were untreated in Arunachal Pradesh compared with more than one third untreated in the economically advanced state of Gujarat (38.2%: 95% CI 35.0 to 41.4). However, only 35.4% (95% CI 32.0 to 38.9) were untreated in the economically poor state of Bihar. Figure 3 shows a negative association between the percentage of untreated conditions and state NSDP. However, a few low-income states, such as Meghalaya and Jammu and Kashmir, had a much lower proportion of untreated conditions.

**Fig. 4:**
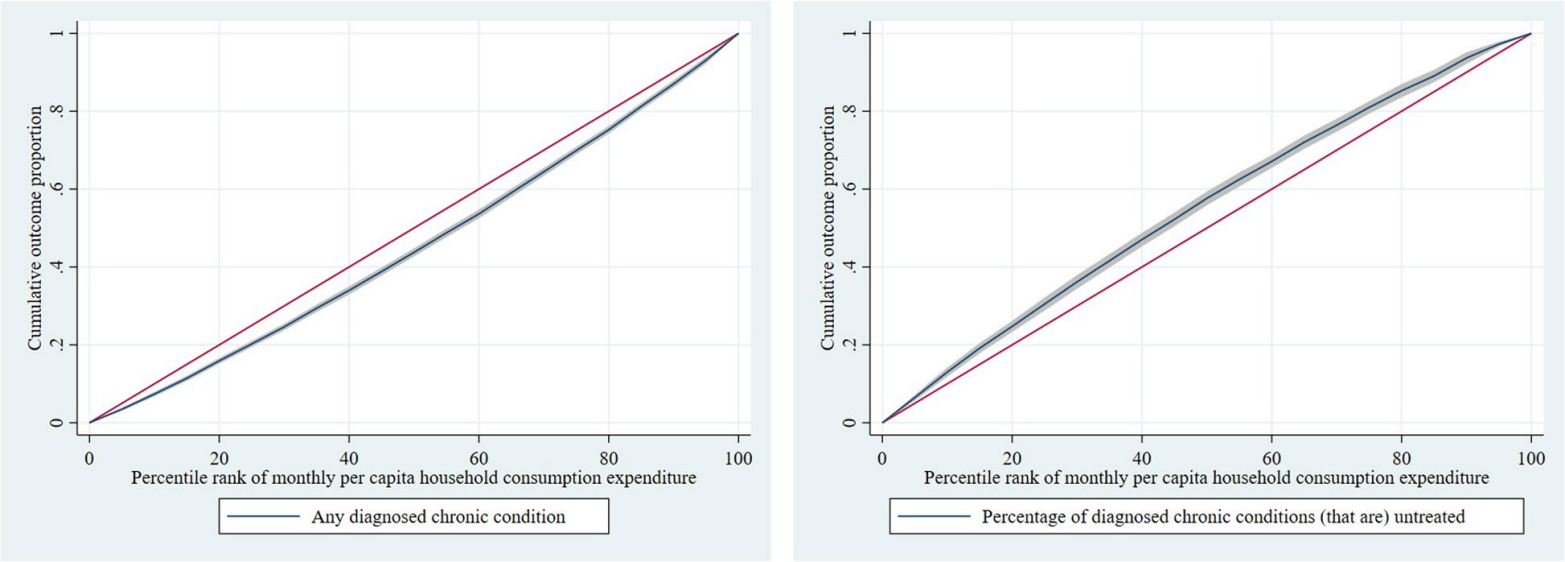
Adjusted concentration curve of any diagnosed chronic condition and untreated fraction of all diagnosed chronic conditions, adults aged 45 years and over in India, LASI 2017–18

The concertation curve for any diagnosed condition lies below the 45-degreee line (Fig. 4), indicating that richer individuals were disproportionately more likely to report a diagnosed chronic condition. This was confirmed by a dominance test and by a positive concentration index of 0.078 (95% CI 0.067 to 0.090) (Table 3). The concentration index is positive for each type of diagnosed condition, although the confidence interval includes zero for lung disease, cholesterol, and neurological problems (Table 3). The concentration index is also positive for each state (Table 5) indicating that richer people are more likely to report diagnosed chronic conditions throughout the country. The concentration curve for the number of conditions was also below the 45-degreee line, indicating that richer individuals disproportionately reported more diagnosed conditions (S1 Figure).

**Table 5:** Adjusted concentration index of any diagnosed chronic condition and untreated fraction of all diagnosed chronic conditions by states, adults aged 45 years and older in India, LASI 2017–18

		**Any diagnosed chronic condition**		**Untreated fraction of all diagnosed chronic conditions**
	***N***	**Concentration index (95% CI)**	***N***	**Concentration index (95% CI)**
**India**	**64,755**	**0.078 (0.067, 0.090)**	**30,017**	**-0.059 (-0.071, -0.047)**
**State/Uts**				
Andaman and Nicobar	1097	0.053 (0.000, 0.106)	622	0.004 (-0.046, 0.053)
Andhra Pradesh	2295	0.024 (-0.008, 0.055)	1303	-0.019 (-0.051, 0.013)
Arunachal Pradesh	979	0.062 (0.000, 0.124)	263	-0.089 (-0.176, -0.002)
Assam	1991	0.082 (0.056, 0.107)	780	-0.054 (-0.087, -0.021)
Bihar	3276	0.060 (0.027, 0.093)	1331	-0.023 (-0.088, 0.041)
Chandigarh	875	0.024 (-0.037, 0.084)	510	-0.038 (-0.075, 0.000)
Chhatisgarh	1834	0.095 (0.061, 0.130)	469	-0.099 (-0.146, -0.051)
Dadra and Nagar Haveli	862	0.147 (0.077, 0.216)	295	-0.064 (-0.140, 0.012)
Daman and Diu	789	0.030 (-0.027, 0.087)	435	-0.021 (-0.088, 0.046)
Delhi	1152	0.020 (-0.017, 0.057)	582	-0.058 (-0.096, -0.021)
Goa	1208	0.071 (0.036, 0.106)	744	-0.010 (-0.038, 0.018)
Gujarat	1983	0.068 (0.040, 0.096)	876	-0.034 (-0.077, 0.009)
Haryana	1728	0.002 (-0.029, 0.034)	878	-0.040 (-0.088, 0.009)
Himachal Pradesh	1224	0.061 (0.025, 0.096)	575	-0.042 (-0.093, 0.010)
Jammu and Kashmir	1472	0.036 (0.006, 0.066)	855	-0.024 (-0.066, 0.019)
Jharkhand	2192	0.110 (0.075, 0.145)	716	-0.076 (-0.108, -0.043)
Karnataka	1997	0.120 (0.066, 0.174)	895	-0.095 (-0.144, -0.046)
Kerala	2171	0.023 (-0.003, 0.049)	1505	0.001 (-0.027, 0.028)
Lakshadweep	996	0.045 (0.010, 0.081)	606	-0.031 (-0.057, -0.005)
Madhya Pradesh	2672	0.053 (0.007, 0.098)	919	-0.024 (-0.072, 0.024)
Maharashtra	3541	0.026 (-0.007, 0.058)	1832	-0.044 (-0.068, -0.019)
Manipur	1240	0.045 (0.012, 0.078)	459	0.013 (-0.028, 0.054)
Meghalaya	877	0.057 (-0.010, 0.124)	253	-0.094 (-0.150, -0.039)
Mizoram	1114	0.085 (0.037, 0.132)	404	-0.054 (-0.109, 0.001)
Nagaland	1192	0.086 (0.017, 0.154)	198	-0.095 (-0.165, -0.025)
Odisha	2583	0.127 (0.093, 0.160)	955	-0.040 (-0.074, -0.006)
Puducherry	1272	0.009 (-0.033, 0.052)	798	-0.087 (-0.134, -0.041)
Punjab	1885	0.010 (-0.017, 0.038)	1128	-0.026 (-0.048, -0.003)
Rajasthan	2097	0.055 (0.024, 0.086)	930	-0.041 (-0.081, 0.000)
Sikkim	974	0.040 (-0.019, 0.099)	490	-0.034 (-0.086, 0.017)
Tamil Nadu	3173	0.040 (0.015, 0.065)	1757	-0.065 (-0.093, -0.037)
Telangana	2147	0.042 (0.011, 0.073)	1184	-0.044 (-0.072, -0.016)
Tripura	1013	0.110 (0.071, 0.149)	453	-0.012 (-0.062, 0.037)
Uttar Pradesh	4219	0.043 (0.021, 0.065)	1472	-0.057 (-0.087, -0.027)
Uttarakhand	1250	0.082 (0.037, 0.127)	594	-0.055 (-0.103, -0.008)
West Bengal	3385	0.041 (0.013, 0.068)	1951	-0.077 (-0.121, -0.033)

The concentration curve for the proportion of untreated conditions lies above the 45-degree line (Fig. 4) – confirmed by a dominance test – which indicates that untreated conditions conditional on having any diagnosed condition were more concentrated among the poorer people. A negative concentration index of -0.059 (95% CI -0.071 to -0.047) confirmed that poorer individuals were more likely to have more untreated conditions (Table 3). Negative concentration indices imply that poorer individuals were more likely to remain untreated for each type of condition, with the difference only being non-significant for neurological problems (Table 3). The concentration index for untreated fraction of all diagnosed chronic conditions was negative for all the states (Table 5), indicating that poorer people were treated for fewer diagnosed conditions throughout the country.

**Fig. 5:**
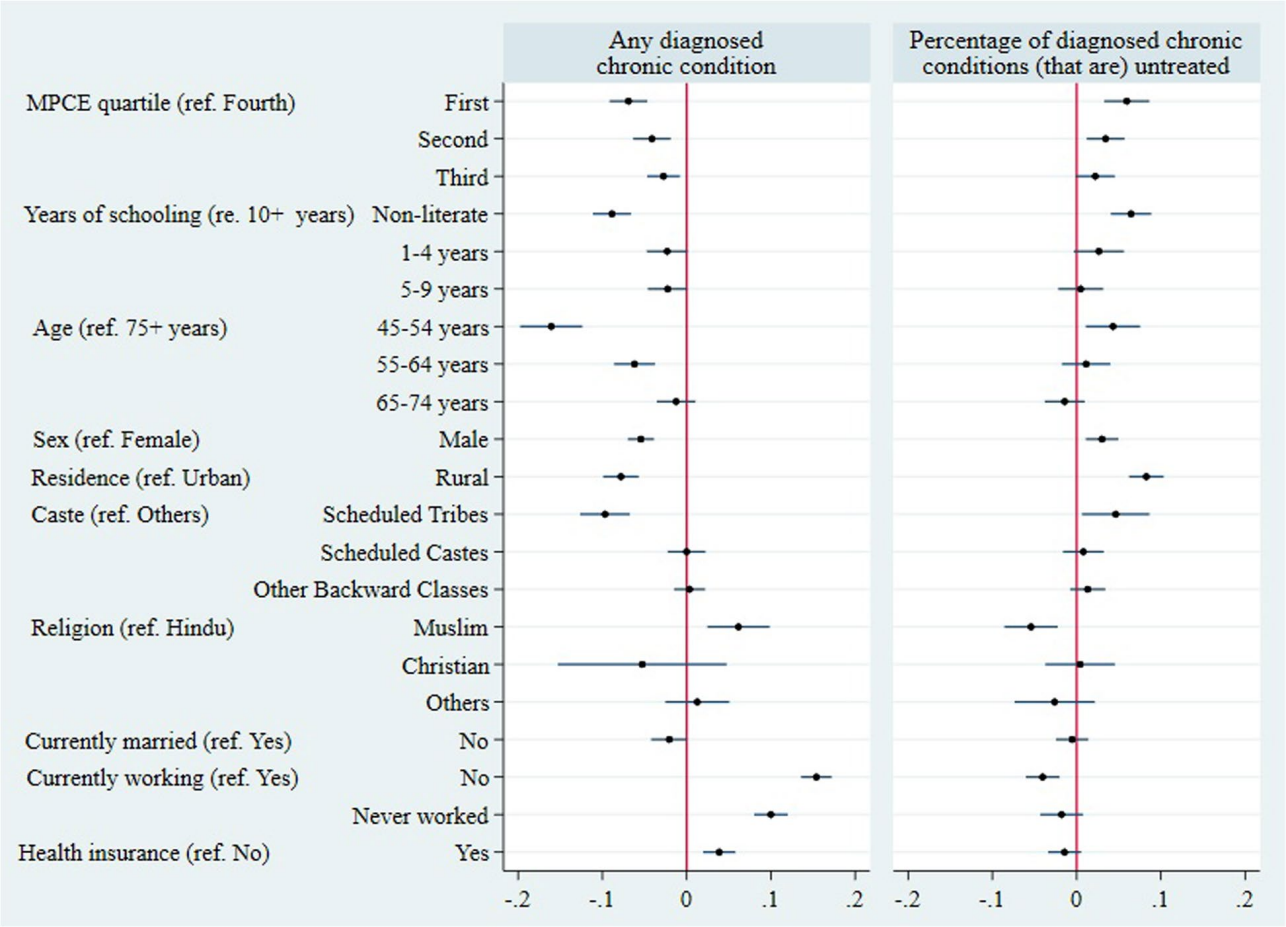
Partial associations of any diagnosed chronic condition and untreated fraction of all diagnosed chronic conditions with covariates, adults aged 45 years and older in India. Notes: Figure shows estimated average marginal effects. See S5 Table for estimates in table format

Estimates of marginal effects obtained from the multivariable probit analysis show that the probability of reporting any diagnosed chronic condition was 6.9 percentage points (pp) (95% CI 4.7 to 9.1) lower in the poorest quartile group compared to the richest group (Fig. 5). Participants having no formal schooling had 8.9 pp (95% CI 6.6 to 11.1) lower probability of reporting a diagnosis of a chronic condition than those with 10 or more years of schooling. Self-reported diagnoses were lower among males, rural dwellers, and members of scheduled tribes.

Marginal effects from the fractional regression analysis of the untreated fraction of all diagnosed chronic conditions indicate that the poorest group had 6.0 pp (95% CI 3.3 to 8.6) more untreated conditions than the richest group. The education gradient in untreated conditions became steeper after controlling for other sociodemographic characteristics and state fixed effects, with a difference of 6.5 pp (95% CI 4.1 to 8.9) between the least and the most educated groups. A significantly higher untreated fraction of all diagnosed chronic conditions was found among younger adults, males, rural dwellers, and members of scheduled tribes compared to their counterparts.

## Discussion

In this paper, we examined the sociodemographic and regional pattern in the any diagnosed chronic conditions and treatment of chronic conditions among adults aged 45 years and above in India. About half of these adults reported having one or more diagnosed chronic conditions. The prevalence of such conditions was higher among females and urban residents, and increased with age and educational attainment, and living standard. These findings are consistent with the literature [41]. The lower prevalence of diagnosed chronic conditions among poorer and less educated individuals is plausible due to higher proportions of undiagnosed conditions in these groups [7, 42, 43]. We also found large variations in diagnosed chronic conditions across the states of India. Prevalence was lower in the north-eastern states of Nagaland and Meghalaya and the poorer states of Chhattisgarh, Madhya Pradesh, and Uttar Pradesh, while it was higher in the more developed states of Kerala and Goa. The high proportion of undiagnosed chronic conditions in the poorer and hilly states of India is possibly due to the lack of health care facilities in those states and the poorer economic conditions of the households. While better public awareness of chronic diseases and more accessible health services are likely reasons for the higher prevalence of diagnosed chronic disease in the more economically developed states. Our analyses revealed that prevalence of self-reported diagnosis was highest for hypertension, followed by arthritis and diabetes and lowest for neurological disorders.

Our findings indicate that more than a quarter of diagnosed chronic conditions were untreated. Absence of treatment was higher among individuals who were poorer, less educated, members of disadvantaged tribes, and resident in rural areas. The higher proportion of untreated chronic conditions experienced by poorer individuals was evident in every state of India, suggesting that the finding of pro-rich bias in treatment is robust.

These findings are consistent with other studies that suggest that economic conditions, level of education, employment status, and place of residence are key determinants of untreated morbidity and preventive care [14, 44]. Lack of health education, inability to afford drugs, non-availability of prescribed medication, perception of adverse impact of medication on normal daily activities, fear of side-effects, forgetfulness, poor family support, and cultural and religious beliefs are among the reasons of variations in treatment-seeking behaviour [45–49]. Care for chronic conditions in India is largely provided by private health care providers, who are often located in city centres, and tends to be expensive [50]. Discontinuation of medication and use of less than the prescribed dose are common challenges in the treatment of chronic conditions. There is some evidence that many patients turn to herbal medicines and spiritual healing as therapeutic alternatives because of their easy affordability [51]. These patterns of health care supply and demand presumably contribute to the observed high prevalence of untreated conditions among poorer and socially disadvantaged populations. We also found that the proportion of untreated chronic conditions conditional on diagnosis declined with age and was higher among females. Health insurance was not associated with a lower proportion of untreated chronic conditions. This is possibly due to the fact that health insurance in India mainly covers hospitalisation rather than treatments for chronic conditions, including maintenance medication.

The socioeconomic inequality in the treatment of each type of chronic condition conditional on diagnosis favoured richer individuals. This socioeconomic gradient was steepest for neurological disorders, about half of which were untreated despite being diagnosed.

The Government of India’s National Programme for Prevention and Control of Cancer, Diabetes, Cardiovascular Diseases and Stroke [52] aims to improve awareness, treatment, and control of major chronic diseases [53]. To achieve this objective, there is an urgent need to increase diagnosis of chronic conditions through screening and public awareness [54]. To reduce the burden of chronic disease burden, a comprehensive plan is needed to generate awareness and screen populations to reduce undiagnosed conditions, and to improve access to preventive care and treatments in order to reduce unmet needs of the diagnosed.

We acknowledge the following limitations of our study. First, the prevalence of any of the chronic diseases may have been underestimated due to the self-reported nature of the cases. Biomarkers were not available for all seven conditions. Their availability would have affected the direction and magnitude of socioeconomic inequality if the underdiagnosed cases were higher among the poor. Such a pattern would exacerbate our finding of higher treatment gaps among less privileged population groups. Second, we did not analyse reasons for non-adherence to treatment due to lack of the requisite data. Discontinuation of medication may have led to the reporting of untreated conditions at the time of the survey.

For conditions, such as hypertension and diabetes, that could be indicated by biomarkers without reported diagnosis, our analysis was limited to the step of a care cascade that conditions on diagnosis and estimates the proportion (not) treated. Our contribution was to examine treatment for seven chronic conditions, including some for which there were no biomarkers and a full cascade of care could not be constructed. By examining many conditions and aggregating over them to estimate the proportion of diagnosed conditions that are untreated, we carried out an analysis of treatment for older Indians with diagnosed chronic conditions that is more comprehensive than care cascade studies done for any one condition.

Monitoring socioeconomic inequalities in the prevalence and treatment of chronic diseases can help reduce their burden. Our findings suggest that improving access to treatment for chronic disease experienced by older adults in India, especially those who are poorer, less educated and living in rural areas, is a major challenge. Improving diagnosis of otherwise undetected chronic disease is not enough. Those who are diagnosed, especially the socially disadvantaged, must be given greatly improved access to inexpensive generic drugs that are known to be highly cost-effective in managing chronic conditions.

## Supplementary Information

**Additional file 1: S1 Table.** Description of self-reported diagnosis of chronic diseases and its treatment. **S2 Table.**  Age-sex adjusted estimates of number of diagnosed chronic conditions by socioeconomic and demographic characteristics among adults 45 years and above in India, LASI 2017-18. **S3 Table.** Age sex adjusted prevalence of diagnosed chronic disease diagnosis among older adults 45+ in India, LASI 2017-18. **S4 Table.** Prevalence of any diagnosed chronic condition and untreated fraction of all diagnosed chronic conditions adults aged 45 years and older across states in India, LASI 2017-18.

## Data Availability

The data set we have analyzed during the current study are available https://iipsindia.ac.in/sites/default/files/LASI_DataRequestForm_0.pdf. The stata code used for the current study can be available from the corresponding author.
