# Supplementary material for: Sociodemographic and geographic inequalities in diagnosis and treatment of older adults’ chronic conditions in India: a nationally representative population-based study
Source: BMC Health Serv Res. 2023 Apr 3;23:332. doi: 10.1186/s12913-023-09318-6 (PMC10069025; doi:10.1186/s12913-023-09318-6)
Supplement: Supplementary file 1 — Additional file 1: S1 Table. Description of self-reported diagnosis of chronic diseases and its treatment. S2 Table. Age-sex adjusted estimates of number of diagnosed chronic conditions by socioeconomic and demographic characteristics among adults 45 years and above in India, LASI 2017-18. S3 Table. Age sex adjusted prevalence of diagnosed chronic disease diagnosis among older adults 45+ in India, LASI 2017-18. S4 Table. Prevalence of any diagnosed chronic condition and untreated fraction of all diagnosed chronic conditions adults aged 45 years and older across states in India, LASI 2017-18. [file 12913_2023_9318_MOESM1_ESM.doc]

Supplementary Material

Inequalities in untreated chronic illness in the middle-aged and older population of India: a nationally representative population-based study

**Table of contents**

| Supplementary Texts | 1 |
| --- | --- |
| Supplementary Tables | 2 |
| Supplementary Figures | 10 |

**S1 Text: Measurement of monthly per capita consumer expenditure (MPCE)**

Monthly per capita consumer expenditure (MPCE) is a direct measure of a household’s living standard. Official estimates of poverty and inequality in India are derived from MPCE measured in the National Sample Survey (NSS). The LASI measure of MPCE followed the NSS methodology (an abridged version of consumption schedule), although not to the same level of detail. Household food consumption in 10 categories of food was reported for a reference period of the last 7 days. The value of food produced by a household for its own consumption and food eaten outside was included. Non-food expenditure was reported for a reference period of the last 30 days for more frequently purchased items and over the last 365 days for seldom-purchased durable goods. We calculated household expenditure for a 30-day period by multiplying reported food consumption by 30/7 and dividing non-food expenditures reported for the last year by 365/30. We excluded from MPCE out-of-pocket expenditure on healthcare and medicines in order to avoid misclassifying households with sickness and medical spending as economically better off. The variables used in derivation of MPCE are similar to that of NSS. After adjusting for the different reference periods and aggregating across categories of expenditure, we divided by household size to get MPCE. In the analysis sample of individuals, we applied sampling weights to get the quintiles of MPCE and used these values to categorize participants into four quartile groups of equal (weighted) size: 1^st^ quartile 25%, 2^nd^ quartile 25%, 3^rd^ quartile 25%, and 4^th^ quartile 25%.

| **S1 Table. Description of self-reported diagnosis of chronic diseases and its treatment** | | |
| --- | --- | --- |
| **Chronic diseases** | **Question on chronic diseases** | **Treatment** |
| Hypertension or high blood pressure | Has any health professional ever told you that you have hypertension or high blood pressure (no, yes)? | 1. In order to control your blood pressure or hypertension, are you currently taking any medication? 2. In order to control your blood pressure, are you under salt or other diet restrictions? |
| Diabetes or high blood sugar | Has any health professional ever told you that you have diabetes or high blood sugar (no, yes)? | 1. In order to treat or control your diabetes or high blood sugar, are you currently taking medications that you swallow? 2. Are you currently using insulin shots/injections? 3. In order to control your diabetes, are you following a special diet? |
| Chronic lung disease | Has any health professional ever told you that you have chronic lung disease such as asthma, chronic obstructive pulmonary disease/chronic bronchitis or other chronic lung problems (no, yes)? | 1. Are you receiving physical or respiratory therapy, or any other treatment for your lung disease? |
| Chronic heart diseases | Has any health professional ever told you that you have chronic heart diseases such as coronary heart disease (heart attack or Myocardial Infarction), congestive heart failure, or other chronic heart problems | 1. Are you currently taking any medication for your heart disease? |
| Stroke | Has any health professional ever told you that you have stroke (no, yes)? | 1. Are you currently taking any medications because of your stroke or its complications? 2. Are you receiving physical or occupational therapy because of your stroke or its complications? |
| Cholesterol | Has any health professional ever told you that you have higher cholesterol (no, yes)? | 1. Do you regularly take medications to help lower your cholesterol? |
| Arthritis | Has any health professional ever told you that you have arthritis or rheumatism, Osteoporosis or other bone/joint diseases (no, yes)? | 1. Are you currently taking any medication or receiving other treatments for your arthritis, rheumatism or osteoporosis? |
| Any neurological, or psychiatric problems | Has any health professional ever told you that you have any neurological, or psychiatric problems such as depression, Alzheimer’s/Dementia, unipolar/bipolar disorders, convulsions, Parkinson’s etc (no, yes)? | 1. Are you currently taking any psychiatric or psychological treatment or therapy for your condition? 2. Are you currently taking tranquilizers, antidepressants, or other types of medication for neurological or psychiatric problem (s)? |

| **S2 Table. Age-sex adjusted estimates of number of diagnosed chronic conditions by socioeconomic and demographic characteristics among adults 45 years and above in India, LASI 2017-18** | |
| --- | --- |
|  | **N=64,755** |
|  | **Average number of diagnosed chronic health conditions** |
| **India** | **0.70 (0.67 , 0.73)** |
| **MPCE Quartile** |  |
| First | 0.54 (0.51 , 0.56) |
| Second | 0.64 (0.61 , 0.66) |
| Third | 0.73 (0.70 , 0.77) |
| Fourth | 0.91 (0.84 , 0.98) |
| P-value | <0.001 |
| **Educational attainment** |  |
| Illiterate | 0.57 (0.55 , 0.60) |
| Less than 5 years | 0.76 (0.72 , 0.80) |
| 5-9 years completed | 0.81 (0.77 , 0.85) |
| 10 years or more | 1.01 (0.88 , 1.11) |
| P-value | <0.001 |
| **Age** |  |
| 45-54 | 0.50 (0.54 ,0. 24) |
| 55-64 | 0.71 (0.75 , 0.17) |
| 65-74 | 0.89 (0.97 , 0.14) |
| 75+ | 0.90 (0.95 , 0.16) |
| P-value | <0.001 |
| **Sex** |  |
| Male | 0.64 (0.62 , 0.67) |
| Female | 0.75 (0.72 , 0.79) |
| P-value | <0.001 |
| **Residence** |  |
| Rural | 0.60 (0.58 , 0.62) |
| Urban | 0.94 (0.88 , 1.01) |
| P-value | <0.001 |
| **Caste** |  |
| Scheduled Tribes | 0.40 (0.36 , 0.44) |
| Scheduled Castes | 0.65 (0.61 , 0.68) |
| OBC | 0.73 (0.68 , 0.78) |
| Others | 0.80 (0.76 , 0.83) |
| P-value | <0.001 |
| **Religion** |  |
| Hindu | 0.68 (0.64 , 0.71) |
| Muslim | 0.86 (0.76 , 0.96) |
| Christian | 0.68 (0.57 , 0.79) |
| Others | 0.77 (0.71 , 0.83) |
| P-value | <0.001 |
| **Marital Status** |  |
| Currently marries | 0.71 (0.68 , 0.74) |
| Currently not married | 0.69 (0.65 , 0.73) |
| P-value | 0.248 |
| **Working status** |  |
| Currently working | 0.52 (0.49 , 0.54) |
| Ever worked but currently not working | 0.86 (0.82 , 0.89) |
| Never worked | 0.84 (0.778 , 0.91) |
| P-value | <0.001 |
| **Health Insurance** |  |
| No | 0.69 (0.66 , 0.72) |
| Yes | 0.76 (0.72 , 0.80) |
| P-value | 0.001 |

| **S3 Table : Age sex adjusted prevalence of diagnosed chronic disease diagnosis among older adults 45+ in India, LASI 2017-18** | | | | | | | |
| --- | --- | --- | --- | --- | --- | --- | --- |
|  | Hypertension | Heart Disease or stroke | Diabetes | Cholesterol | Arthritis | Respiratory Disease | Any Neurological problems |
| **India** | 27.3 (26.2 , 28.2) | 5.5 (5.0 , 5.9) | 12.1 (11.1 , 13.1) | 2.3 (2.0 , 2.5) | 16.1 (15.2 , 16.9) | 6.7 (5.9 , 7.4) | 2.4 (2.1 , 2.7) |
| **MPCE Quartile** |  |  |  |  |  |  |  |
| First | 20.2 (19.0 , 21.3) | 4.0 (3.5 , 4.6) | 7.6 (6.7 , 8.4) | 0.9 (0.7 , 1.2) | 14.1 (12.9 , 15.2) | 5.7 (5.2 , 6.3) | 2.3 (1.7 , 2.8) |
| Second | 24.9 (23.7 , 26.1) | 5.1 (4.4 , 5.8) | 8.6 (7.9 , 9.4) | 1.5 (1.3 , 1.8) | 16.1 (15.0 , 17.3) | 7.2 (6.3 , 8.0) | 2.2 (1.8 , 2.5) |
| Third | 29.7 (28.5 , 31.0) | 5.7 (5.1 , 6.2) | 12.6 (11.4 , 13.8) | 2.2 (1.7 , 2.6) | 16.7 (15.5 , 17.8) | 6.1 (5.6 , 6.7) | 2.7 (2.3 , 3.2) |
| Fourth | 35.4 (33.9 , 36.9) | 7.1 (5.9 , 8.4) | 19.6 (17.1 , 22.1) | 4.5 (3.9 , 5.0) | 17.5 (15.4 , 19.6) | 7.7 (5.1 , 10.2) | 2.6 (2.1 , 3.0) |
| F , statistic (p , value) | 100.47 (<0.001) | 9.96 (<0.001) | 41.59 (<0.001) | <0.001 | 5.59(<0.001) | 2.95 (0.0315) | 1.41 (0.237) |
| **Educational attainment** |  |  |  |  |  |  |  |
| Illiterate | 21.8 (20.9 , 22.7) | 4.0 (3.6 , 4.4) | 7.4 (6.4 , 8.4) | 0.9 (0.7 , 1.1) | 14.9 (14.1 , 15.8) | 6.3 (5.8 , 6.8) | 2.2 (1.8 , 2.6) |
| Less than 5 years | 29.1 (27.4 , 30.8) | 6.0 (4.9 , 7.0) | 13.1 (11.8 , 14.4) | 2.6 (2.0 , 3.2) | 18.9 (17.3 , 20.5) | 7.3 (6.2 , 8.3) | 2.7 (2.1 , 3.4) |
| 5 , 9 years completed | 32.3 (31.0 , 33.7) | 6.6 (5.8 , 7.5) | 14.7 (13.0 , 16.5) | 3.2 (2.8 , 3.7) | 16.7 (15.4 , 18.0) | 7.2 (6.3 , 8.0) | 2.9 (2.4 , 3.4) |
| 10 years or more | 39.2 (37.6 , 40.8) | 8.5 (6.5 , 10.5) | 23.8 (21.5 , 26.0) | 5.7 (4.9 , 6.5) | 17.2 (14.2 , 20.3) | 6.8 (2.9 , 10.8) | 2.3 (1.7 , 3.0) |
| F , statistic (p , value) | 149.33 (<0.001) | 10.16 (<0.001) | 75.92 (<0.001) | <0.001 | 7.17 (<0.001) | 1.58 (0.175) | 1.92 (0.124) |
| **Age** |  |  |  |  |  |  |  |
| 45 , 54 | 20.6 (19.5 , 21.7) | 2.8 (2.4 , 3.2) | 9.3 (7.2 , 11.4) | 1.9 (1.7 , 2.2) | 11.4 (10.6 , 12.3) | 3.8 (3.4 , 4.2) | 2.1 (1.7 , 2.5) |
| 55 , 64 | 27.4 (26.3 , 28.5) | 5.1 (4.6 , 5.6) | 13.2 (12.1 , 14.3) | 2.5 (2.2 , 2.9) | 16.4 (15.0 , 17.9) | 6.8 (5.4 , 8.2) | 2.2 (1.8 , 2.5) |
| 65 , 74 | 34.1 (32.6 , 35.5) | 8.3 (6.6 , 10.1) | 15.0 (13.1 , 16.8) | 2.4 (1.8 , 3.0) | 20.4 (18.6 , 22.2) | 8.9 (7.2 , 10.7) | 2.4 (2.0 , 2.8) |
| 75+ | 35.0 (33.3 , 36.7) | 8.4 (6.5 , 10.2) | 11.7 (10.3 , 13.0) | 2.2 (1.1 , 3.4) | 20.4 (18.4 , 22.4) | 10.4 (9.2 , 11.7) | 4.1 (2.9 , 5.3) |
| F , statistic (p , value) | 62.50(<0.001) | 30.36 (<0.001) | 6.55 (<0.001) | <0.001 | 45.50 (<0.001) | 42.61 (<0.001) | 3.87 (0.009) |
| **Sex** |  |  |  |  |  |  |  |
| Male | 23.4 (22.5 , 24.3) | 6.2 (5.6 , 6.7) | 12.3 (11.3 , 13.4) | 2.2 (1.9 , 2.6) | 12.8 (11.9 , 13.8) | 7.2 (6.4 , 8.1) | 2.3 (2.1 , 2.6) |
| Female | 31.0 (30.1 , 32.0) | 4.8 (4.1 , 5.6) | 11.8 (10.6 , 13.0) | 2.3 (2.0 , 2.6) | 18.8 (17.8 , 19.9) | 6.2 (5.4 , 7.0) | 2.5 (2.1 , 2.9) |
| F , statistic (p , value) | 207.29 (<0.001) | 6.76 (0.009) | 0.86 (0.397) | <0.001 | 150.13 (<0.001) | 8.25 (0.004) | 0.56 (0.453) |
| **Residence** |  |  |  |  |  |  |  |
| Rural | 22.9 (22.1 , 23.7) | 4.5 (4.1 , 4.9) | 8.1 (7.6 , 8.5) | 1.3 (1.1 , 1.5) | 15.7 (14.9 , 16.6) | 6.5 (6.1 , 6.9) | 2.4 (2.0 , 2.7) |
| Urban | 37.8 (36.4 , 39.2) | 7.7 (6.7 , 8.7) | 21.4 (19.2 , 23.6) | 4.5 (3.9 , 5.1) | 16.8 (15.0 , 18.7) | 7.1 (5.0 , 9.2) | 2.6 (2.1 , 3.1) |
| F , statistic (p , value) | 327.79 (<0.001) | 34.87 (<0.001) | 141.43 (<0.001) | <0.001 | 1.14 (0.264) | 0.36 (0.507) | 0.40 (0.5261) |
| **Caste** |  |  |  |  |  |  |  |
| Scheduled Tribes | 16.0 (14.0 , 17.9) | 2.3 (1.7 , 2.8) | 5.1 (4.2 , 6.1) | 0.7 (0.4 , 1.0) | 10.2 (8.7 , 11.6) | 4.8 (3.9 , 5.8) | 1.5 (1.0 , 2.1) |
| Scheduled Castes | 24.5 (23.1 , 25.9) | 5.3 (4.5 , 6.0) | 8.8 (7.7 , 9.9) | 1.5 (0.9 , 2.1) | 15.9 (14.5 , 17.3) | 7.1 (6.3 , 8.0) | 2.6 (1.7 , 3.6) |
| OBC | 27.3 (26.2 , 28.4) | 5.3 (4.5 , 6.1) | 13.6 (11.8 , 15.4) | 2.2 (1.9 , 2.5) | 16.7 (15.4 , 18.1) | 7.1 (5.7 , 8.5) | 2.4 (2.0 , 2.8) |
| Others | 33.4 (32.0 , 34.7) | 6.8 (6.3 , 7.4) | 14.1 (13.1 , 15.1) | 3.5 (3.0 , 3.9) | 16.9 (15.7 , 18.1) | 6.2 (5.5 , 6.8) | 2.6 (2.1 , 3.0) |
| F , statistic (p , value) | 81.03 (<0.001) | 44.11 (<0.001) | 66.22 (<0.001) | <0.001 | 20.94 (<0.001) | 4.59 (0.004) | 3.35 (0.018) |
| **Religion** |  |  |  |  |  |  |  |
| Hindu | 26.1 (25.3 , 27.0) | 5.2 (4.7 , 5.7) | 11.4 (10.5 , 12.3) | 2.0 (1.7 , 2.2) | 16.0 (15.1 , 17.0) | 6.6 (5.8 , 7.5) | 2.4 (2.1 , 2.8) |
| Muslim | 33.7 (30.9 , 36.5) | 7.2 (6.0 , 8.3) | 16.3 (10.7 , 21.8) | 2.8 (2.1 , 3.6) | 17.4 (15.4 , 19.5) | 7.2 (6.0 , 8.3) | 2.5 (1.8 , 3.2) |
| Christian | 31.1 (27.5 , 34.8) | 5.7 (3.4 , 7.9) | 14.9 (11.7 , 18.1) | 3.8 (2.3 , 5.2) | 12.9 (10.7 , 15.2) | 6.1 (4.5 , 7.7) | 2.7 (1.7 , 3.7) |
| Others | 36.7 (33.2 , 40.2) | 6.2 (4.7 , 7.6) | 12.7 (10.8 , 14.5) | 6.2 (4.3 , 8.2) | 14.7 (12.5 , 17.0) | 6.5 (4.7 , 8.2) | 1.7 (1.1 , 2.4) |
| F , statistic (p , value) | 20.65(<0.001) | 3.57 (<0.01) | 2.45 (0.061) | <0.001 | 2.87 (0.031) | 0.49 (0.690) | 1.46 (0.224) |
| **Marital Status** |  |  |  |  |  |  |  |
| Currently marries | 27.3 (26.4 , 28.3) | 5.6 (5.1 , 6.0) | 12.6 (11.4 , 13.7) | 2.4 (2.1 , 2.7) | 16.4 (15.5 , 17.3) | 6.5 (5.9 , 7.1) | 2.4 (2.0 , 2.7) |
| Currently not married | 27.9 (26.7 , 29.1) | 5.2 (4.2 , 6.2) | 10.8 (9.7 , 11.9) | 1.9 (1.5 , 2.4) | 15.3 (14.0 , 16.6) | 7.0 (5.7 , 8.4) | 2.6 (2.2 , 3.0) |
| F , statistic (p , value) | 0.72 (0.713) | 0.43 (0.515) | 8.24 (0.003) | 0.0963 | 2.29 (0.120) | 0.94 (0.332) | 0.75 (0.386) |
| **Working status** |  |  |  |  |  |  |  |
| Currently working | 20.5 (19.6 , 21.4) | 2.9 (2.6 , 3.2) | 8.0 (7.1 , 8.8) | 1.4 (1.2 , 1.5) | 13.9 (12.9 , 15.0) | 5.0 (4.2 , 5.8) | 1.6 (1.3 , 1.9) |
| Ever worked but currently not working | 32.3 (31.0 , 33.5) | 7.6 (6.9 , 8.3) | 15.3 (13.4 , 17.1) | 2.5 (2.1 , 2.9) | 18.9 (17.7 , 20.0) | 8.4 (7.6 , 9.3) | 3.2 (2.7 , 3.7) |
| Never worked | 33.7 (32.2 , 35.3) | 7.8 (6.3 , 9.4) | 16.8 (14.8 , 18.8) | 4.0 (3.3 , 4.7) | 16.2 (14.8 , 17.6) | 7.4 (5.7 , 9.2) | 3.2 (2.5 , 4.0) |
| F , statistic (p , value) | 197.60 (<0.001) | 88.26 (<0.001) | 113.77 (<0.001) | <0.001 | 23.85 (<0.001) | 27.58 (<0.001) | 17.15 (<0.001) |
| **Health Insurance** |  |  |  |  |  |  |  |
| No | 27.2 (26.3 , 28.0) | 5.4 (4.9 , 5.9) | 11.7 (10.7 , 12.6) | 2.1 (1.9 , 2.4) | 15.5 (14.5 , 16.5) | 6.7 (5.8 , 7.6) | 2.4 (2.1 , 2.7) |
| Yes | 28.7 (27.4 , 30.1) | 5.7 (5.1 , 6.4) | 13.7 (11.8 , 15.6) | 2.8 (2.1 , 3.4) | 18.3 (16.9 , 19.7) | 6.5 (5.7 , 7.4) | 2.7 (2.0 , 3.4) |
| F , statistic (p , value) | 4.38 (0.021) | 0.72 (0.445) | 5.46 (0.022) | 0.0685 | 9.47 (0.003) | 0.04 (0.834) | 0.56 (0.453) |

| **S4 Table. Prevalence of any diagnosed chronic condition and untreated fraction of all diagnosed chronic conditions adults aged 45 years and older across states in India, LASI 2017-18** | | | | | |
| --- | --- | --- | --- | --- | --- |
|  |  | **Prevalence of any diagnosed chronic condition** |  |  | **Untreated fraction of all diagnosed chronic conditions** |
|  | **N** | **(95% CI), %** |  | **N** | **(95% CI), %** |
| India | **64,755** | **46.1 (44.9 , 47.3)** |  | **30,017** | **27.5 (26.2 , 28.7)** |
| State/UTs |  |  |  |  |  |
| Andaman and Nicobar | 1244 | 60.9 (55.7 , 66.0) |  | 622 | 19.6 (15.5 , 23.7) |
| Andhra Pradesh | 2,679 | 58.3 (55.5 , 61.2) |  | 1303 | 20.3 (17.0 , 23.6) |
| Arunachal Pradesh | 1,215 | 35.2 (27.9 , 42.5) |  | 263 | 51.8 (39.6 , 64.0) |
| Assam | 2,366 | 42.3 (39.4 , 45.2) |  | 780 | 21.3 (18.4 , 24.2) |
| Bihar | 3,520 | 39.4 (35.7 , 43.0) |  | 1331 | 35.4 (32.0 , 38.9) |
| Chandigarh | 1,026 | 59.2 (53.7 , 64.6) |  | 510 | 12.8 (10.0 , 15.7) |
| Chhatisgarh | 2,055 | 27.6 (24.0 , 31.2) |  | 469 | 27.1 (21.8 , 32.5) |
| Dadra and Nagar Haveli | 1,090 | 41.8 (35.2 , 48.4) |  | 295 | 35.7 (29.7 , 41.8) |
| Daman and Diu | 991 | 55.9 (51.4 , 60.4) |  | 435 | 27.0 (23.0 , 31.1) |
| Delhi | 1319 | 54.1 (51.4 , 56.8) |  | 582 | 14.7 (10.5 , 18.9) |
| Goa | 1,427 | 61.9 (58.2 , 65.7) |  | 744 | 11.9 (9.5 , 14.3) |
| Gujarat | 2,341 | 45.2 (41.2 , 49.2) |  | 876 | 38.2 (35.0 , 41.4) |
| Haryana | 1,898 | 52.0 (48.2 , 55.7) |  | 878 | 27.2 (23.0 , 31.5) |
| Himachal Pradesh | 1,388 | 48.1 (44.1 , 52.1) |  | 575 | 28.3 (24.9 , 31.7) |
| Jammu and Kashmir | 1,613 | 58.3 (54.3 , 62.4) |  | 855 | 17.4 (13.5 , 21.3) |
| Jharkhand | 2,464 | 33.7 (30.8 , 36.6) |  | 716 | 25.3 (22.0 , 28.6) |
| Karnataka | 2,420 | 47.2 (41.0 , 53.3) |  | 895 | 22.6 (17.0 , 28.2) |
| Kerala | 2497 | 69.4 (66.5 , 72.2) |  | 1505 | 22.2 (19.4 , 25.0) |
| Lakshadweep | 1,139 | 60.1 (55.7 , 64.4) |  | 606 | 15.0 (11.0 , 18.9) |
| Madhya Pradesh | 2,914 | 34.3 (30.7 , 37.8) |  | 919 | 33.7 (29.2 , 38.2) |
| Maharashtra | 3,973 | 51.0 (47.6 , 54.5) |  | 1832 | 22.8 (20.5 , 25.1) |
| Manipur | 1369 | 37.6 (32.2 , 43.1) |  | 459 | 24.7 (20.2 , 29.2) |
| Meghalaya | 969 | 31.7 (24.4 , 38.9) |  | 253 | 20.1 (12.6 , 27.5) |
| Mizoram | 1246 | 37.3 (32.3 , 42.3) |  | 404 | 42.8 (36.1 , 49.5) |
| Nagaland | 1,316 | 21.6 (14.9 , 28.3) |  | 198 | 32.8 (24.4 , 41.2) |
| Odisha | 2917 | 35.9 (31.7 , 40.1) |  | 955 | 29.4 (25.5 , 33.4) |
| Puducherry | 1428 | 58.7 (55.1 , 62.2) |  | 798 | 20.6 (15.8 , 25.5) |
| Punjab | 2124 | 60.7 (57.8 , 63.6) |  | 1128 | 23.0 (21.0 , 25.0) |
| Rajasthan | 2,244 | 44.7 (41.3 , 48.0) |  | 930 | 29.1 (26.2 , 31.9) |
| Sikkim | 1,146 | 54.8 (50.1 , 59.6) |  | 490 | 20.3 (13.3 , 27.2) |
| Tamil Nadu | 3530 | 54.2 (51.1 , 57.3) |  | 1757 | 28.4 (25.5 , 31.3) |
| Telangana | 2475 | 56.4 (53.3 , 59.5) |  | 1184 | 24.8 (21.4 , 28.1) |
| Tripura | 1,195 | 47.6 (42.2 , 52.9) |  | 453 | 30.1 (25.3 , 34.9) |
| Uttar Pradesh | 4567 | 34.3 (32.1 , 36.5) |  | 1472 | 31.6 (28.3 , 35.0) |
| Uttarakhand | 1,358 | 46.5 (41.1 , 51.8) |  | 594 | 28.6 (23.8 , 33.3) |
| West Bengal | 3,933 | 57.9 (54.5 , 61.3) |  | 1951 | 29.8 (25.6 , 34.1) |

| **S5 Table. Partial associations of any diagnosed chronic condition and untreated fraction of diagnosed chronic conditions with covariates adults aged 45 years and older in India** | | | |
| --- | --- | --- | --- |
|  | **N=64,755** |  | **N=30,017** |
|  | **Any diagnosed chronic condition** |  | **Untreated fraction of diagnosed chronic conditions** |
|  | **Average marginal effect (95% CI)** |  | **Average marginal effect (95% CI)** |
| **MPCE Quartile** |  |  |  |
| First | -0.069 (-0.091 , -0.047) |  | 0.060 (0.033 , 0.086) |
| Second | -0.041 (-0.064 , -0.019) |  | 0.034 (0.012 , 0.057) |
| Third | -0.027 (-0.047 , -0.008) |  | 0.022 (-0.001 , 0.045) |
| Fourth | ref |  | ref |
| **Educational attainment** |  |  |  |
| Illiterate | -0.089 (-0.111 , -0.066) |  | 0.065 (0.041 , 0.089) |
| Less than 5 years | -0.023 (-0.047 , 0.001) |  | 0.026 (-0.003 , 0.056) |
| 5-9 years completed | -0.023 (-0.046 , 0.001) |  | 0.005 (-0.022 , 0.032) |
| 10 years or more | ref |  | ref |
| **Age** |  |  |  |
| 45-54 | -0.161 (-0.198 , -0.124) |  | 0.043 (0.011 , 0.075) |
| 55-64 | -0.062 (-0.086 , -0.038) |  | 0.011 (-0.018 , 0.040) |
| 65-74 | -0.012 (-0.035 , 0.010) |  | -0.014 (-0.038 , 0.010) |
| 75+ | ref |  | ref |
| **Sex** |  |  |  |
| Male | -0.054 (-0.070 , -0.039) |  | 0.030 (0.011 , 0.050) |
| Female | ref |  | ref |
| **Residence** |  |  |  |
| Rural | -0.078 (-0.099 , -0.057) |  | 0.083 (0.062 , 0.103) |
| Urban | ref |  | ref |
| **Caste** |  |  |  |
| Scheduled Tribes | -0.097 (-0.126 , -0.067) |  | 0.046 (0.006 , 0.086) |
| Scheduled Castes | 0.000 (-0.022 , 0.022) |  | 0.008 (-0.016 , 0.032) |
| OBC | 0.004 (-0.015 , 0.022) |  | 0.013 (-0.008 , 0.034) |
| Others | ref |  | ref |
| **Religion** |  |  |  |
| Hindu | ref |  | ref |
| Muslim | 0.062 (0.025 , 0.099) |  | -0.054 (-0.086 , -0.022) |
| Christian | -0.053 (-0.153 , 0.047) |  | 0.004 (-0.037 , 0.045) |
| Others | 0.013 (-0.025 , 0.051) |  | -0.026 (-0.074 , 0.022) |
| **Marital Status** |  |  |  |
| Currently married | ref |  | ref |
| Currently not married | -0.021 (-0.042 , 0.001) |  | -0.005 (-0.025 , 0.014) |
| **Working status** |  |  |  |
| Currently working | ref |  | ref |
| Ever worked but currently not working | 0.154 (0.136 , 0.172) |  | -0.040 (-0.060 , -0.020) |
| Never worked | 0.100 (0.080 , 0.120) |  | -0.018 (-0.043 , 0.007) |
| **Health Insurance** |  |  |  |
| No | ref |  | ref |
| Yes | 0.039 (0.020 , 0.058) |  | -0.014 (-0.034 , 0.005) |

**S1 Figure. Adjusted concentration curve of number of diagnosed health conditions, adults aged 45 years and older** in India, LASI 2017-18

| 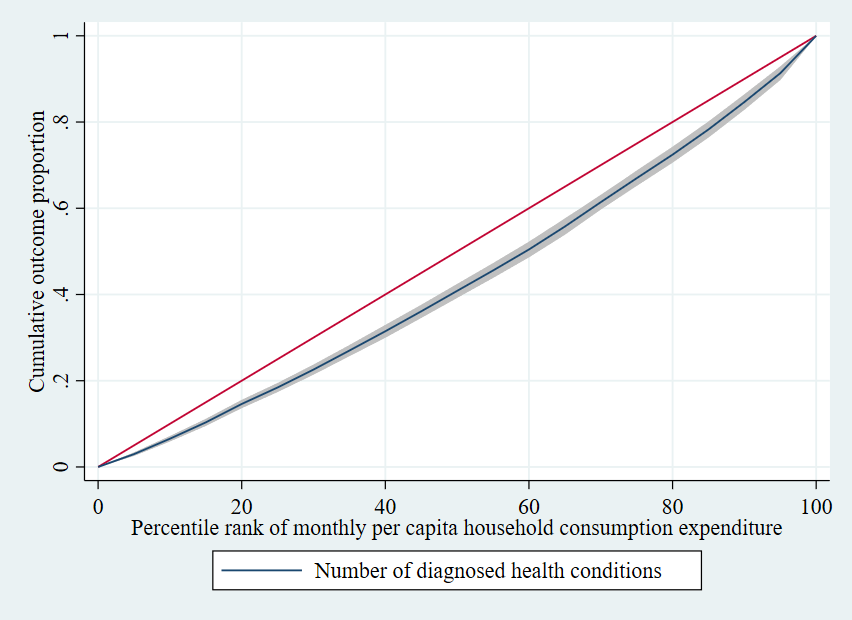 |
| --- |
